# Can Reproductive Health Voucher Programs Improve Quality of Postnatal Care? A Quasi-Experimental Evaluation of Kenya’s Safe Motherhood Voucher Scheme

**DOI:** 10.1371/journal.pone.0122828

**Published:** 2015-04-02

**Authors:** Claire Watt, Timothy Abuya, Charlotte E. Warren, Francis Obare, Lucy Kanya, Ben Bellows

**Affiliations:** 1 Jacaranda Health, Nairobi, Kenya; 2 Population Council, Nairobi, Kenya; 3 Population Council, Washington DC, United States of America; University of Missouri-Kansas City, UNITED STATES

## Abstract

This study tests the group-level causal relationship between the expansion of Kenya’s Safe Motherhood voucher program and changes in quality of postnatal care (PNC) provided at voucher-contracted facilities. We compare facilities accredited since program inception in 2006 (phase I) and facilities accredited since 2010-2011 (phase II) relative to comparable non-voucher facilities. PNC quality is assessed using observed clinical content processes, as well as client-reported outcome measures. Two-tailed unpaired t-tests are used to identify differences in mean process quality scores and client-reported outcome measures, comparing changes between intervention and comparison groups at the 2010 and 2012 data collection periods. Difference-in-differences analysis is used to estimate the reproductive health (RH) voucher program’s causal effect on quality of care by exploiting group-level differences between voucher-accredited and non-accredited facilities in 2010 and 2012. Participation in the voucher scheme since 2006 significantly improves overall quality of postnatal care by 39% (p=0.02), where quality is defined as the observable processes or components of service provision that occur during a PNC consultation. Program participation since phase I is estimated to improve the quality of observed maternal postnatal care by 86% (p=0.02), with the largest quality improvements in counseling on family planning methods (IRR 5.0; p=0.01) and return to fertility (IRR 2.6; p=0.01). Despite improvements in maternal aspects of PNC, we find a high proportion of mothers who seek PNC are not being checked by any provider after delivery. Additional strategies will be necessary to standardize provision of packaged postnatal interventions to both mother and newborn. This study addresses an important gap in the existing RH literature by using a strong evaluation design to assess RH voucher program effectiveness on quality improvement.

## Introduction

Healthcare interventions delivered in the critical first days after childbirth have the highest potential to prevent maternal and neonatal deaths [1–5]. An estimated 10–27% of the 4 million annual newborn deaths could be averted if effective postnatal care (PNC) were scaled up to 90% coverage globally [1]. PNC can improve maternal and newborn health by timely identifying and addressing maternal postnatal complications, connecting mothers to family planning (FP) services, promoting breastfeeding and immunizations, and increasing access to other key interventions for newborn survival [3, 6]. Unfortunately, PNC coverage remains low in the countries that account for 95% of the global burden of maternal and child mortality, with only one-third of mothers and newborns receiving skilled care in the postnatal period [7–9]. Kenya’s progress on coverage of skilled birth attendance and PNC over the past two decades remains relatively low [10–11]. 56% of Kenya’s deliveries continue to occur at home, with only an estimated 1% of these deliveries occurring under the care of skilled birth attendant [12]. In more than 4 out of 5 of these home deliveries, the mother and newborn do not receive any skilled PNC [12].

Recognizing the need for increasing health care access to mothers and newborns during and after delivery, the Government of Kenya is implementing an output-based approach (OBA), which is an innovative health financing strategy that addresses both supply- and demand-side elements to reduce financial barriers to accessing key health services. Individuals purchase and redeem vouchers for a range of pre-specified health services at a participating health facility of their choice at subsidized cost. On the demand side, voucher programs are designed to improve access among disadvantaged populations by reducing out-of-pocket health expenditures through use of targeted service subsidies. By targeting specific demographic and high-risk groups, such as low-income mothers, vouchers improve population health [13–15]. On the supply side, competition for voucher reimbursements across the public and private sectors can improve operational efficiency and stimulate quality improvement in service provision [15]. Facilities are required to undergo an accreditation process prior to participation in the voucher scheme, and are also subject to ongoing quality assessments. This ensures a minimum standard of quality of care, and may incentivize facilities to improve service quality in order to participate [16]. An additional mechanism for quality improvement is the reimbursement strategy, through which participating facilities are paid based on number of voucher clients and type of care provided to each. Competition for this pool of clients, many of whom could not previously afford facility-based maternal or infant health care, may further stimulate improvements in the quality of service provision [15–16].

A 2011 systematic review identified only five high-quality evaluations of reproductive health (RH) voucher programs [17]. The strongest evidence is regarding increased utilization of antenatal care (ANC), institutional delivery, PNC, and contraceptive uptake as a result of voucher program implementation [18–23]. While quality improvement is a stated goal of many voucher programs, evidence is limited [17]. Bangladesh’s RH voucher program was found to improve several dimensions of client-reported quality of care, including client receipt of contraceptives and self-reported satisfaction [22, 24–25]. Most existing studies on quality improvement are based on before-and-after without comparison group or cross-sectional designs [17]. In this paper, we compare PNC quality before and after voucher program expansion in facilities in both voucher and non-voucher sites. We test the statistical significance of plausible group-level causal relationships between the expansion of Kenya’s Safe Motherhood voucher program and changes in the quality of PNC at voucher-contracted facilities.

### Kenya’s reproductive health voucher program

Kenya’s RH voucher program includes three vouchers that respectively subsidize FP, gender-based violence recovery, and maternal health services [13, 26]. For a highly subsidized price equivalent to $2.50, eligible women are able to buy a “Safe Motherhood” voucher that can be redeemed for up to four ANC visits, delivery (including Caesarean section and treatment of obstetric complications), and one PNC visit up to 6 weeks after delivery at participating, quality-accredited public or private health facilities. Vouchers are exclusively available to low-income women, identified through a poverty-grading tool administered to prospective clients. Facilities receive an average reimbursement of $119 per Safe Motherhood voucher client [26].

The parastatal National Coordinating Agency on Population and Development first implemented the voucher program in 2006 in 54 facilities across the three districts of Kisumu, Kitui, and Kiambu, as well as in two informal settlements in Nairobi (Phase I). An additional 25 facilities in the original districts and 14 facilities in Kilifi county were added in Phase II (2010–2011), during which time the country adopted a new constitution that created 47 semi-federated county governments from the former nationally-administered provinces and districts [18]. In 2010, the Ministry of Health took over the project and subsequently launched Phase III (2012–2015). As of 2014, there are 226 contracted facilities in the five counties that were created from the eponymous sub counties. Largely financed by the German Development Bank, $8.4 million was invested in the program in Phase I, with an additional $13 million scheduled for Phase II [27]. There is additional support from Kenya with a budgetary line item for OBA services as a flagship project within the Ministry of Health. Between the RH voucher program’s 2006 start and March 2011, the Safe Motherhood voucher subsidized an estimated 96,000 institutional deliveries; this figure is expected to increase to 120,000 deliveries by the end of 2014 [26].

### Conceptual framework for assessing quality of postnatal care

**Fig 1:**
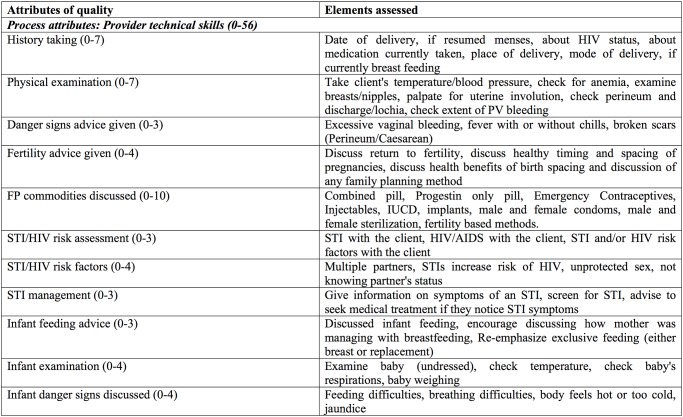
Attributes of Postnatal Care: Structure, Process, and Outcome.

We assess quality of care using the framework proposed by Donabedian and quality assessment principles described by Bruce [28–29]. Quality of care is framed here based on two domains of quality, process and outcome. Process indicators are defined as specific and observable elements of interactions between clients and providers during a PNC visit. Outcomes indicators comprise a number of measures to assess PNC clients’ experiences and perceptions of quality of care. Fig 1 presents the technical and interpersonal processes and health outcomes used to evaluate quality of care provided during a postnatal consultation, based on the World Health Organization’s guidelines for PNC [30].

Process and outcome attributes of quality were assessed using data from observed provider-client interactions and client exit interviews, respectively. Process indicators include technical attributes of maternal and newborn care and interpersonal relations on the part of the health provider. These process attributes are added with equal weights to create a summative quality process score with a maximum of 64 points. Summary indices of clinical process have been advocated for in the context of measuring the quality of ANC, as such measures capture the clinical content of care provided rather than simple receipt of a consultation [31]. Five self-reported outcome measures of quality of PNC were also assessed, including whether or not both mother and newborn received any postnatal checkup, timing of checkups in relation to delivery, and self-reported satisfaction with PNC services. We acknowledge that client-reported satisfaction reflects expectations and does not necessarily correlate with high-quality care; however, if it is assumed that these limitations remain constant over time, changes in self-reported satisfaction at various phases of program implementation are a useful outcome for assessing client-centered aspects of quality.

## Methods

### Study Design

**Fig 2:**
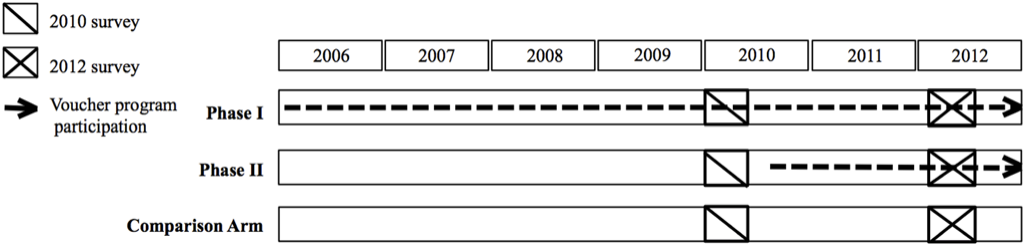
Timing of Surveys and Voucher Program Rollout.

The study uses a quasi-experimental design to evaluate the impact of the Kenyan OBA voucher scheme on increasing access to, and quality of, selected reproductive health services by comparing voucher-accredited health facilities with non-voucher facilities in counties with similar characteristics (defined later). Data were collected at health facilities in 2010 and 2012. Fig 2 describes data collection activities. Comparison and intervention facilities were sampled from different counties in order to improve the validity of the comparison group by minimizing facility and client selection effects that would be expected to arise if comparison facilities were sampled from voucher counties.

Phase I comprised facilities participating since the program’s inception in 2006; these facilities had participated in the program for at least 4 years at the time of the first-round of data collection in 2010. Phase II is comprised of voucher facilities that began participating in the voucher scheme in 2010–2011. The 2010 data therefore represents a true pre-intervention baseline only for the Phase II voucher facilities. Quality of PNC was assessed using 2010 and 2012 data from 19 randomly selected 2006 voucher-accredited health facilities in four counties, 16 randomly selected 2010/2011 voucher-accredited health facilities in three counties, and 18 non-voucher health facilities in three comparable counties [13]. Comparison facilities were selected using a pair-wise matching sampling design; for each sampled treatment facility, a non-voucher facility in a comparison district with similar characteristics in 2010 was sampled. Facility matching characteristics included facility level, sector, staffing levels and types, urban/rural location, fees, and average client profile [32]. The evaluation design is described elsewhere [13, 32].

### Data collection

We conducted analyses of quantitative data collected from 53 health facilities (34 public, 19 private, non-governmental, or faith-based) in eight counties in Kenya. Health facility assessments obtained PNC client information through observations of client-provider interactions of postnatal consultations matched with client exit interviews with observed postnatal clients. During the 2010 round of data collection, a total of 394 PNC clients were observed in the 2006-accredited voucher facilities, 310 clients at 2010/2011-accredited voucher facilities, and 230 clients at comparison facilities, for a total of 934 observations in 2010. In 2012, 259 PNC observations were used for the 2006-accredited voucher facilities, 169 clients for 2010/2011-accredited voucher facilities, and 141 clients at comparison facilities, for a total of 569 post-rollout observations.

### Data analysis

We test for differences in quality improvements in PNC provision between voucher-accredited facilities and comparable non-voucher facilities. Quality of PNC is first evaluated using a composite score based on the observed technical and interpersonal content of a PNC consultation and client-reported outcomes data. Pearson’s chi-square tests were used to evaluate differences in distribution of facility characteristics and self-reported client socioeconomic status. Statistical significance of differences in client-reported outcome measures, which include receipt of maternal and newborn postnatal checkup, timing of maternal and newborn first checkup relative to delivery, and client-reported satisfaction, were evaluated using two-tailed unpaired t-tests with unequal variance. Two-tailed unpaired t-tests with unequal variance were also used to evaluate group differences in mean process quality scores comparing intervention and comparison groups.

Difference-in-differences (DD) analysis is used to estimate the voucher program’s causal effect by exploiting group-level differences across two or more dimensions. The DD estimator equals the average change in outcomes in the treatment group, after the average change in outcomes in the comparison group is subtracted. The DD approach adjusts for time invariant differences between the two groups, as well as time-varying influences affecting both groups equivalently. The difference-in-differences approach to isolating program effect rests upon the usual assumptions of Ordinary Least Squares (OLS), as well as the additional identification assumption of parallel trends: Internal validity rests upon the premise that changes in PNC quality over time in the group of comparison facilities are equivalent to the changes in PNC quality over time that would have been observed in the intervention facilities, had the voucher program not been implemented.

DD estimators for process outcomes are estimated using negative binomial models, with each of the 16 individual and summative process quality score outcomes modeled individually. Negative binomial models are appropriate for modeling dependent variables within a discrete, restricted range, and were selected to account for over-dispersion of the count (score) process outcomes. Negative binomial estimates are presented as incidence rate ratios. We additionally report estimates using the OLS estimator in S1 Table as a robustness check. DD estimators of program impact on the dichotomous outcome measures were estimated using logistic regression models. DD estimates are shown for three model specifications, the first including data sampling time (2010 vs. 2012 post-rollout) and treatment type (phase I or phase II, vs. comparison) in addition to the DD estimator, the second adding facility- and client-level covariates, and the final adding facility-level fixed effects. Covariates in the latter two model specifications include facility type, facility sector, and mean client socioeconomic status (by quintile). Facility type is defined categorically by level, with the lowest level comprised of dispensaries, nursing homes, and clinics, the second level health centers, and the third level hospitals and sub-district hospitals. Facility sector is defined as public, non-governmental, private, or mission/faith-based. Individual-level client socioeconomic status is included as a ordinal variable in models 2 and 3, as the voucher program limits eligibility for vouchers to individuals below a specific poverty threshold. Client-level socioeconomic quintiles were generated using principal components analysis of the following household characteristics and assets: source of drinking water, toilet type, cooking fuel type, electricity, radio, television, telephone, refrigerator, solar power, lantern, bicycle, motorcycle, car/truck, boat with motor, boat without motor, and animal- or human-drawn cart. Estimates from all three models are presented to assess sensitivity of the results to model specification. Standard errors are clustered at the health facility level, the unit of the intervention.

The general difference-in-differences model can be described as:

$$y_{if}=\beta_{0}+\beta_{1}P_{f}+\beta_{2}T_{f}+\beta_{3}P_{f}T_{f}+X_{i}\gamma +Z_{f}\delta +\varepsilon_{if}$$

where T_f_ is a dummy variable for voucher accreditation status of facility *f*, P_f_ is a dummy variable representing intervention time (2010 or 2012), X_i_ is a vector of individual-level covariates, Z_f_ is a vector of facility-level covariates, and *β*_3_ is the difference-in-difference estimator, the OLS coefficient for the interaction of being in the voucher facility group at the post-rollout time period. *y*_*if*_ represents the processes and outcome indicators, each modeled separately. *y*_*if*_ is therefore the 16 individual and summative process scores and 5 PNC outcome indicators that were examined as dependent variables of PNC quality for individual *i* at facility *f*.

Outcome variables were specified prior to analysis according to the definition of postnatal care quality presented in Fig 1. Pre-specification of the outcomes of interest is one method to reduce the probability of finding statistical significance by chance when multiple outcomes are being tested. A second method is to report p-values adjusted for the total number of hypotheses tested. In addition to cluster-adjusted p-values, we present q-values adjusted for the false detection rate (FDR), which is the likelihood of finding a statistically significant effect by chance for any one of the individual outcomes in the group of outcomes being tested. False detection rate-adjusted q-values were calculated using the method proposed by Benjamini, Krieger, and Yekutieli (2006) and operationalized by Anderson (2008) [33–34]. Analyses were performed using Stata software, version 12.1 (StataCorp, College Station, TX).

### Ethics Statement

Ethical approval for the evaluation was granted by Population Council’s institutional review board (IRB) No. 470 and the Kenya Medical Research Institute (KEMRI) SCC 174. Informed consent was obtained prior to all observations of PNC consultations and client exit interviews. Interviews were conducted in settings that ensured privacy and confidentiality. Data collectors received training on ethical conduct prior to data collection. All participants provided their written informed consent prior to participation in the survey, with one copy of the written consent retained by the research team and one copy retained by the participant.

## Results

Mean characteristics of the 2010 sample at the facility- and client-level are presented in Table 1. Sampled clients in phase I and phase II facilities in 2010 were significantly less likely than sampled clients in the comparison group to receive PNC at higher-level facilities. Clients observed at phase I facilities were also significantly more likely to be at private facilities, relative to the comparison arm (p<0.001). No significant differences in facility sector were observed between the phase II and comparison samples. We find no differences across the phase I and comparison samples in client socioeconomic status; however, the pool of clients sampled in phase II facilities in 2010 were significantly poorer than the comparison sample (p<0.001). As the political and economic center of the country, Nairobi has no reasonable comparison county that can serve as its counterfactual. As a robustness check, we present descriptive statistics and estimates of program impact with samples both including and excluding Nairobi.

**Table 1:** Facility- and Individual-Level Characteristics of Observed PNC Consultations at 2010 Data Collection.

					p-values		
Facility characteristics [Observations (percent)]	Phase I facilities	Phase I facilities excl. Nairobi	Phase II facilities	Comparison facilities	Phase I vs. Comparison	Phase I excl. Nairobi vs. Comparison	Phase II vs. Comparison
**Total sampled facilities**	19	14	16	17			
***Facility type***							
Higher-level (hospital/sub-district hospital)	10	8	6	10			
Lower-level (health center, dispensary, clinic or nursing home)	9	6	10	7			
***Facility sector***							
Public (government)	8	7	13	12			
Private (non-governmental, private, or mission/faith-based)	11	7	3	5			
**PNC Client Observations**							
***Facility type***							
Higher-level (hospital/sub-district hospital)	226 (57.4%)	201 (63.01%)	146 (47.1%)	157 (68.3%)	0.01	0.20	<0.01
Lower-level (health center, dispensary, clinic or nursing home)	168 (42.6%)	118 (37.0%)	164 (52.9%)	73 (31.7%)			
***Facility sector***							
Public (government)	190 (48.2%)	183 (57.4%)	237 (76.5%)	170 (73.9%)	<0.01	<0.01	0.50
Private (non-governmental, private, or mission/faith-based)	204 (51.8%)	136 (42.6%)	73 (23.6%)	60 (26.1%)			
***Client Socioeconomic status***							
Poor (lowest three wealth quintiles)	223 (56.6%)	200 (62.7%)	242 (78.1%)	131 (57.0%)	0.93	0.18	<0.01
Less poor (highest two wealth quintiles)	171 (43.4%)	119 (37.3%)	68 (21.9%)	99 (43.0%)			

**Notes:** p-values generated using Pearson’s *χ* ^2^ tests for independence.

When Nairobi facilities were excluded, facility level differences between the phase I and comparison samples become statistically insignificant. Nevertheless, the 2010 differences in client samples stratified by study arm strengthen the rationale for adjusting for facility sector, level, and client socioeconomic status in estimates of program effect.

### Observed postnatal care processes

**Fig 3:**
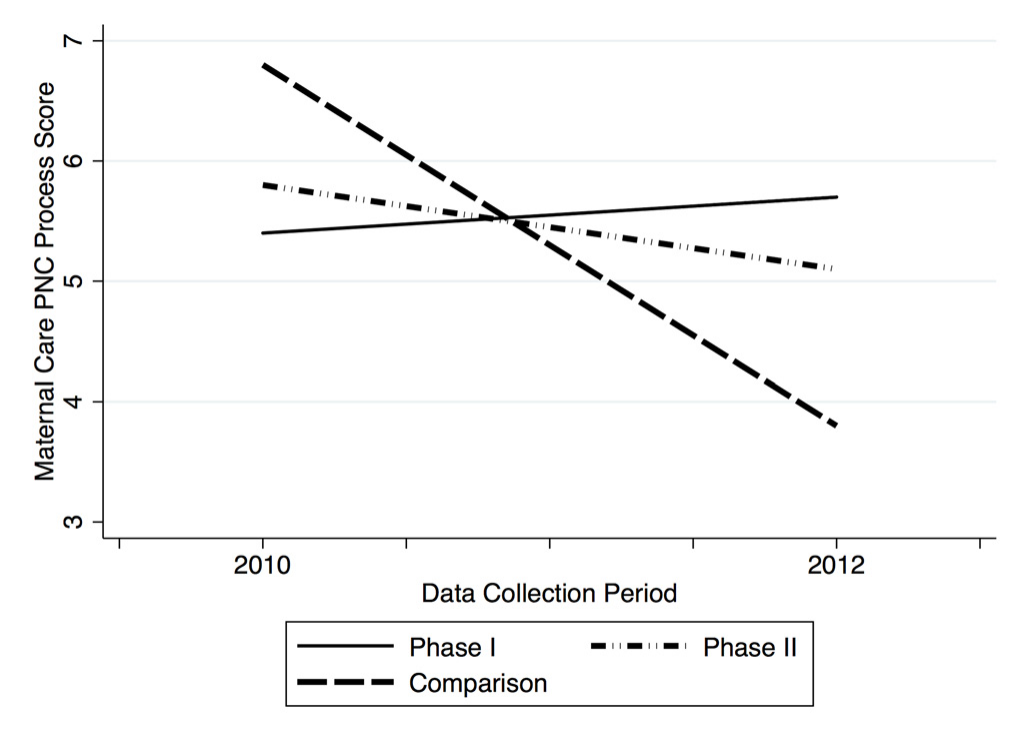
Maternal PNC process scores, by study arm and time.

**Fig 4:**
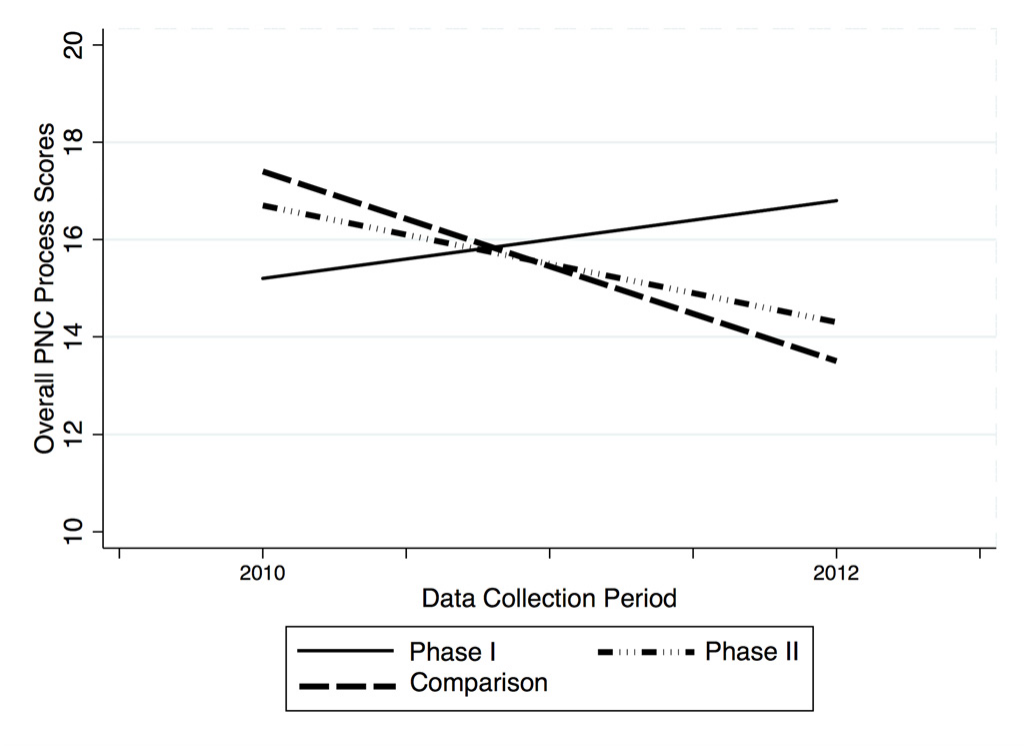
Overall PNC process scores, by study arm and time.

Individual and overall process scores for each intervention arm and the comparison facilities at 2010 and 2012 (post-rollout), as well as the magnitude and statistical significance of differences between groups at each time point, are presented in Table 2. Difference-in-differences estimates of phase I and phase II voucher program impacts on key process indicators are presented in Table 2, Columns (1) through (3). Table 3 reports the statistical significance of the key process results adjusted for multiple hypothesis testing. Changes in process scores by group and data collection period are presented graphically in Figs 3 and 4.

**Table 2:** Difference-in-Differences Estimates of Program Effect on PNC Observed Processes.

	****Phase I vs. Comparison Group****								
	****Mean PNC Quality Scores****						****Difference-in-Differences Estimates****		
	****2010 [Mean quality score (SD)]****			****2012 Post-Rollout [Mean quality score (SD)]****			****Arm I**** * ****Post (SE)****		
**Maternal care**	Phase I	Comp-arison	Diff.	Phase I	Comp-arison	Diff.	**(1)**	**(2)**	**(3)**
History taking practices (0–7)	2.3 (1.8)	2.8 (1.8)	0.5*** (0.1)	2.0 (1.8)	1.6 (1.5)	-0.3* (0.2)	1.48* (0.3)	1.47* (0.3)	1.42* (0.3)
Mother physical examination (0–7)	1.2 (1.8)	1.3 (1.9)	0.1 (0.2)	1.2 (1.6)	0.8 (1.4)	-0.4** (0.2)	1.59 (0.6)	1.51 (0.6)	1.35 (0.6)
Maternal danger signs advice (0–3)	0.2 (0.5)	0.2 (0.5)	0.0 (0.0)	0.2 (0.5)	0.1 (0.5)	-0.1 (0.1)	1.60 (1.3)	1.53 (1.2)	1.93 (1.5)
Fertility advice (0–4)	0.7 (1.0)	1.1 (1.3)	0.4*** (0.1)	1.0 (1.3)	0.6 (1.1)	-0.4*** (0.1)	2.64** (1.0)	2.58** (1.0)	2.54** (1.0)
Family planning methods discussed (0–10)	0.7 (1.9)	0.9 (2.0)	0.2 (0.2)	1.0 (2.1)	0.4 (1.2)	-0.6*** (0.2)	3.40** (1.7)	5.01*** (2.9)	2.34 (1.9)
STI/HIV risk assessment (0–3)	0.2 (0.6)	0.3 (0.7)	0.08 (0.1)	0.20 (0.6)	0.1 (0.4)	-0.09* (0.1)	3.45 (3.2)	3.81 (3.4)	3.59 (3.3)
STI/HIV risk factors (0–4)	0.2 (0.7)	0.17 (0.5)	-0.01 (0.0)	0.2 (0.6)	0.0 (0.3)	-0.1*** (0.0)	2.52 (1.8)	2.53 (1.6)	1.89 (1.4)
STI management (0–3)	0.0 (0.2)	0.0 (0.2)	0.00 (0.0)	0.0 (0.2)	0.0 (0.1)	-0.0 (0.0)	3.36 (4.4)	3.44 (4.3)	4.73 (6.9)
**Total for maternal care (0–41)**	5.4 (5.5)	6.8 (5.8)	1.4*** (0.5)	5.7 (5.9)	3.8 (4.3)	-2.0*** (0.5)	1.92** (0.6)	1.86** (0.5)	1.80* (0.6)
**Newborn care**									
Newborn feeding advice (0–3)	1.5 (1.3)	2.0 (1.3)	0.5*** (0.1)	1.7 (1.2)	1.5 (1.3)	-0.2 (0.1)	1.52 (0.4)	1.46 (0.4)	1.41 (0.4)
Newborn examination (0–4)	1.0 (0.9)	1.0 (0.8)	-0.1 (0.1)	1.3 (1.2)	1 (0.9)	-0.3*** (0.1)	1.20 (0.3)	1.17 (0.3)	1.07 (0.2)
Newborn danger signs advice (0–4)	0.3 (0.7)	0.4 (0.9)	0.2** (0.1)	0.5 (1.1)	0.5 (1.1)	0.0 (0.1)	1.80 (1.1)	1.05 (0.6)	1.31 (1.0)
Documentation (0–4)	3.0 (1.1)	2.9 (1.4)	-0.10 (0.1)	3.4 (1.0)	3.0 (1.5)	-0.5*** (0.1)	1.11 (0.2)	1.13 (0.2)	1.07 (0.2)
**Total for newborn care (0–15)**	5.7 (2.6)	6.2 (2.7)	0.5** (0.2)	6.9 (3.1)	5.9 (3.2)	-1.0*** (0.3)	1.27 (0.2)	1.24 (0.2)	1.19 (0.2)
**Interpersonal skills**									
Creation of rapport (0–8)	4.1 (1.5)	4.4 (2.0)	0.4** (0.2)	4.2 (1.8)	3.8 (1.8)	-0.4** (0.2)	1.20* (0.1)	1.20** (0.1)	1.17 (0.1)
**Overall (0–64)**	**15.2 (7.8)**	**17.4 (8.8)**	**2.2** *** **(0.7)**	**16.8 (9.0)**	**13.5 (7.4)**	**-3.4** *** **(0.8)**	**1.43** ** **(0.2)**	**1.39** ** **(0.2)**	**1.37** * **(0.2)**
Observations	394	230		259	141		1024	1024	1024

*** p<0.01,

** p<0.05,

* p<0.1

Notes: p-values generated using unpaired t-tests assuming unequal variance. “2010” p-values indicate probability of differences between the intervention vs. comparison mean scores at the 2010 data collection time point; “post-rollout” p-values compare the intervention vs. comparison mean sores at the 2012 data collection time point. Difference-in-differences estimates are reported as incidence rate ratios. Robust standard errors are clustered at the health facility level. Columns (1)–(3) report difference-in-difference estimates of program effect under 3 model specifications. Covariates in models (2) and (3) include categorical variables for facility type, facility sector, and client socioeconomic status quintile. The “phase I” covariate is a dummy for facility inclusion in phase I of the voucher program. “Post” is a time dummy for 2012, with the referent group observations from 2010. The DD estimator for columns (1)–(3) is the interaction between the phase I and post dummies.

**Table 3:** Difference-in-Differences Estimates of Program Effect on Phase II Observed PNC processes.

	****Phase II vs. Comparison Group****								
	****Mean PNC Quality Scores****						****Difference-in-Differences Estimates****		
	****2010 [Mean quality score (SD)]****			****2012 Post-Rollout [Mean quality score (SD)]****			****Phase II**** * ****Post (SE)****		
**Maternal care**	Phase II	Comp-arison	Diff.	Phase II	Comp-arison	Diff.	**(1)**	**(2)**	**(3)**
History taking practices (0–7)	1.8 (1.9)	2.8 (1.8)	-1.0*** (0.2)	1.7 (1.7)	1.6 (1.5)	0.1 (0.2)	1.64** (0.3)	1.61** (0.3)	1.43* (0.3)
Maternal physical examination (0–7)	1 (1.9)	1.3 (1.9)	-0.3** (0.2)	0.4 (1.2)	0.8 (1.4)	-0.4** (0.1)	0.73 (0.4)	0.69 (0.4)	0.48 (0.3)
Maternal danger signs advice (0–3)	0.3 (0.8)	0.2 (0.5)	0.1** (0.1)	0.1 (0.4)	0.1 (0.5)	0.0 (0.0)	0.50 (0.3)	0.49 (0.3)	0.59 (0.4)
Fertility advice (0–4)	0.9 (1.3)	1.1 (1.3)	-0.2* (0.1)	0.8 (1.4)	0.6 (1.1)	0.2 (0.1)	1.62 (0.8)	1.56 (0.7)	1.37 (0.7)
Family planning methods discussed (0–10)	1.1 (2.4)	0.9 (2.0)	0.3 (0.2)	1.5 (2.6)	0.4 (1.2)	1.1*** (0.2)	2.74* (1.4)	2.30 (1.17)	0.88 (0.6)
STI/HIV risk assessment (0–3)	0.3 (0.7)	0.3 (0.7)	0.0 (0.1)	0.4 (0.8)	0.1 (0.4)	0.2*** (0.1)	4.27 (4.1)	4.52 (4.29)	3.93 (3.3)
STI/HIV risk factors (0–4)	0.2 (0.7)	0.2 (0.5)	0.0 (0.1)	0.2 (0.7)	0.0 (0.3)	0.2*** (0.1)	3.01 (2.1)	2.82 (1.86)	2.39 (1.5)
STI management (0–3)	0.2 (0.6)	0.0 (0.2)	0.1*** (0.0)	0.0 (0.2)	0.0 (0.1)	0.0 (0.0)	0.59 (0.6)	0.77 (0.83)	0.79 (0.9)
**Total for maternal care (0–41)**	5.8 (7.8)	6.8 (5.8)	-1.02* (0.6)	5.1 (6.3)	3.8 (4.3)	1.38** (0.6)	1.61 (0.5)	1.49 (0.44)	1.19 (0.4)
**Newborn care**									
Newborn feeding advice (0–3)	1.4 (1.4)	2.0 (1.3)	-0.6*** (0.1)	1.2 (1.4)	1.5 (1.3)	-0.30* (0.2)	1.153 (0.5)	1.16 (0.4)	1.2 (0.5)
Newborn examination (0–4)	1.3 (0.9)	1.0 (0.8)	0.4*** (0.1)	1.1 (0.9)	1 (0.9)	0.13 (0.1)	0.83 (0.2)	0.83 (0.2)	0.76 (0.2)
Newborn danger signs advice (0–4)	0.6 (1.2)	0.4 (0.9)	0.2* (0.1)	0.2 (0.6)	0.5 (1.1)	-0.3*** (0.1)	0.25** (0.2)	0.19** (0.1)	0.20** (0.2)
Documentation (0–4)	3.3 (1.1)	2.9 (1.4)	0.5*** (0.1)	3.2 (1.1)	3.0 (1.5)	0.25* (0.2)	0.93 (0.1)	0.93 (0.1)	0.87 (0.1)
**Total for newborn care (0–15)**	6.5 (3.2)	6.2 (2.7)	0.4 (0.3)	5.7 (2.7)	5.9 (3.2)	-0.21 (0.3)	0.91 (0.2)	0.92 (0.2)	0.89 (0.1)
**Interpersonal skills**									
Creation of rapport (0–8)	4.2 (2.3)	4.4 (2.0)	-0.1 (0.2)	3.4 (1.7)	3.8 (1.8)	-0.4* (0.2)	0.93 (0.2)	0.89 (0.2)	0.85 (0.1)
**Overall (0–64)**	**16.7 (11.6)**	**17.4 (8.9)**	**-0.7 (0.9)**	**14.3 (9.6)**	**13.5 (7.4)**	**0.8 (1.0)**	**1.11 (0.2)**	**1.09 (0.2)**	**1.03 (0.2)**
Observations	310	230		169	141		850	850	850

*** p<0.01,

** p<0.05,

* p<0.1

Notes: p-values generated using unpaired t-tests assuming unequal variance. “2010” p-values indicate probability of differences between the intervention vs. comparison mean scores at the 2010 data collection time point; “post-rollout” p-values compare the intervention vs. comparison mean sores at the 2012 data collection time point. Difference-in-differences estimates are reported as incidence rate ratios. Robust standard errors are clustered at the health facility level. Columns (1)–(3) report difference-in-difference estimates of program effect under 3 model specifications. Covariates in models (2) and (3) include categorical variables for facility type, facility sector, and client socioeconomic status quintile. The “phase I” covariate is a dummy for facility inclusion in phase I of the voucher program. “Post” is a time dummy for 2012, with the referent group observations from 2010. The DD estimator is the interaction between the phase II and post dummies.

#### Phase I

Quality improvements in the phase I group were observed in each PNC domain: maternal care, newborn care, and interpersonal skills. In 2010, phase I facilities scored significantly lower than comparison facilities on each process dimension and overall care. By the post-rollout period (2012) phase I facilities performed significantly higher on average on every dimension individually and overall in terms of PNC processes as compared to the comparison facilities. This switch appears to be driven both by slight improvements in scores from 2010 to the post-rollout period in the intervention facilities, as well as by decreasing scores in the comparison facilities.

The difference-in-difference estimates of program impact attribute an increase in overall quality of PNC processes to participation in the voucher program since 2006 (phase I) of 39% (p = 0.03) under the preferred model (2), which adjusts for individual- and facility-level covariates. The program’s impact on quality appears concentrated in the maternal care dimension, with phase I program participation estimated to improve overall maternal health quality scores by 86% (False discovery rate adjusted q = 0.06). The largest quality improvements were found in counseling on contraceptive method choice (IRR 5.0; FDR q = 0.04) and return to fertility (IRR 2.6; FDR q = 0.04). There is little variation in the estimated coefficients on overall process scores between the crude model (Column 1), the preferred model, which includes facility- and individual-level covariates (Column 2), and the fixed effects model (Column 3). False discovery rate-adjusted q- values are presented in Table 4. Results from the goodness of fit tests for key process outcomes can be found in S1 Table.

**Table 4:** False Discovery Rate-Adjusted Q-Values.

	Phase I vs. Comparison Group			Phase II vs. Comparison Group		
	Phase I*Post + covariates	Cluster-adjusted p-value	FDRq-values	Phase II*Post + covariates	Cluster-adjusted p-value	FDRq-values
History taking practices	1.47	0.06	0.13	1.61	0.02	0.20
Maternal physical exam	1.51	0.26	0.28	0.69	0.49	0.39
Maternal danger signs advice	1.53	0.57	0.31	0.49	0.30	0.33
Fertility advice	2.58	0.01	0.04	1.56	0.35	0.33
Family planning methods discussion	5.01	0.01	0.04	2.30	0.10	0.28
STI/HIV risk assessment	3.81	0.13	0.18	4.52	0.11	0.28
STI/HIV risk factors	2.53	0.15	0.18	2.82	0.12	0.28
STI management	3.44	0.32	0.30	0.77	0.81	0.68
Overall maternal care score	1.86	0.02	0.06	1.49	0.18	>0.99
Overall newborn care score	1.24	0.15	0.08	0.92	0.56	>0.99
Interpersonal care score	1.20	0.05	0.06	0.89	0.50	>0.99

Notes: False discovery rate-adjusted q-values for individual process scores within the maternal care domain account for increased false discovery rates due to the 8 outcomes tested within the overall maternal care score (considering phase I and phase II separately). FWER-adjusted p-values for the overall domains (overall maternal care, overall newborn care, and interpersonal care scores) account for the 3 domain outcomes. FDR q-values were calculated using the sharpened two-stage procedure proposed by Anderson in Anderson (2008), "Multiple Inference and Gender Differences in the Effects of Early Intervention: A Reevaluation of the Abecedarian, Perry Preschool, and Early Training Projects", Journal of the American Statistical Association, 103(484), 1481–1495 and available at: http://are.berkeley.edu/~mlanderson/ARE_Website/Research.html.

#### Phase II

In contrast to the phase I group, the phase II group’s mean scores on maternal care, newborn care, interpersonal skills, and overall quality decreased over the study period. Difference-in-differences estimates of program effect are positive in magnitude overall and in the domain of maternal care, as quality in these domains declined less in the phase II group relative to the comparison group. While estimated improvement in the domain of maternal care attributed to program impact is subjectively similar in magnitude to the estimate in phase I, the estimated phase II program impact is not statistically significant at the α = 0.1 significance level. Likewise, quality improvements in history taking are not found to be statistically significant after adjustment for the false discovery rate. Phase II program participation is not associated with statistically significant quality changes in the newborn care or interpersonal skills domains, or on overall clinical processes.

### Client-reported postnatal care outcomes

Comparisons of PNC outcome measures in phase I and comparison facilities in 2010 and 2012 (post-rollout) are presented in Table 5, with difference-in-differences estimates of phase I and phase II voucher program impact on key process indicators presented in Columns (1)—(3). Goodness of fit results for the preferred model (2) are presented in S1 Table.

**Table 5:** Difference-in-Differences Estimates of Program Effect on Phase I PNC Client-Reported Outcomes.

	****Phase I vs. Comparison Group****								
	****Mean PNC Quality Outcomes****						****Difference-in-Differences Estimates****		
	****2010 [Percentage (SD)]****			****2012 Post-Rollout [Percentage (SD)]****			****Arm I**** * ****Post (SE)****		
****Percent (SD)****	Phase I	Comp-arison	Diff.	Phase I	Comp-arison	Diff.	****(1)****	****(2)****	****(3)****
**Maternal outcomes**									
Mother received any postnatal checkup	56.6% (2.5%)	58.7% (3.3%)	-2.1% (4.1%)	73.4% (2.8%)	71.4% (3.8%)	1.9% (4.7%)	1.20 (0.6)	1.22 (0.6)	1.29 (0.8)
Mothers who received checkup were seen within 48 hours	81.1% (2.6%)	80.7% (3.4%)	0.3% (4.3%)	85.8% (2.5%)	77.0% (4.2%)	8.8%* (4.9%)	1.76 (1.2)	1.701 (1.1)	1.33 (0.9)
**Newborn outcomes**									
Newborn received any postnatal checkup	96.3% (0.9%)	96.9% (1.2%)	-0.52% (1.5%)	98.8% (0.7%)	99.3% (0.7%)	-0.5% (1.0%)	0.72 (1.0)	0.798 (1.076)	1.35 (1.9)
Newborns who received checkup were seen within 48 hours	80.5% (2.1%)	70.8% (3.1%)	9.7%*** (3.7%)	84.2% (2.3%)	67.2% (4.0%)	17.0%*** (4.6%)	1.53 (0.7)	1.397 (0.7)	1.74 (1.0)
**Satisfaction Outcomes**									
Satisfied with services	88.7% (1.6%)	85.2% (2.4%	3.59% (2.8%)	95.0% (1.4%)	87.1% (2.8%)	7.84%** (3.1%)	2.03 (1.2)	2.08 (1.2)	2.205 (1.4)`

*** p<0.01,

** p<0.05,

* p<0.1

Notes: p-values generated using unpaired t-tests assuming unequal variance. “2010” p-values indicate probability of differences between the intervention vs. comparison mean scores at the 2010 data collection time point; “post-rollout” p-values compare the intervention vs. comparison mean sores at the 2012 data collection time point. Difference-in-differences estimates are reported as incidence rate ratios. Robust standard errors are clustered at the health facility level. Columns (1)–(3) report difference-in-difference estimates of program effect under 3 model specifications. Covariates in models (2) and (3) include categorical variables for facility type, facility sector, and client socioeconomic status quintile.

#### Phase I

Coverage of maternal checkups improved in both groups, with an increase in coverage of 16.8% points and 12.7% points in phase I and the comparison arm, respectively. Despite these improvements, nearly a quarter of women reported receiving no postnatal checkup at all at 2012 data collection. By contrast, nearly all newborns were checked after birth in both groups in both 2010 and 2012. There appears to be no significant effect of participating in phase I of the voucher program on the likelihood of mother or newborn receiving a postnatal checkup. Neither does there appear to be a significant program effect on the likelihood of mothers or newborns who did receive checkups being checked in the critical first 48 hours after delivery. While clients at phase I facilities had 2.1 times the odds of reporting complete satisfaction with the PNC services they received, relative to clients in comparison facilities, this estimated effect was not statistically significant at the α = 0.05 level.

#### Phase II

The percentage of women who received any checkup after delivery was not significantly different between the phase II and comparison groups in 2010, at 51% and 59%, respectively; by 2012, a significantly higher percentage of women at comparison facilities (71%) received a checkup, as compared to phase II facilities (54%) (p<0.001). Nearly all newborns in phase II received a checkup at both time points, with no significant differences as compared to the comparison group. Difference-in-differences estimates of phase II voucher program impact on PNC outcomes are presented in Table 6, Columns (1) through (3). We find no statistically significant phase II program effect on the likelihood of mothers or newborns receiving a postnatal checkup. Program participation also does not appear to have improved the likelihood of a checkup occurring within the first 48 hours after delivery among mothers who received any checkup. However, the program appears to have significantly increased the likelihood of newborns who received a checkup being seen within 48 hours of birth (OR = 2.3; p = 0.07). Participation in phase II of the voucher program is also estimated to have significantly increased the odds of full satisfaction by 2.9 times (p = 0.06) under the preferred model (2). As in the PNC process estimates, difference-in-differences estimates of program impact on key PNC outcomes remain subjectively insensitive to model specification.

**Table 6:** Difference-in-Differences Estimates of Program Effect on Phase II PNC Client-Reported Outcomes.

	**Phase II vs. Comparison Group**								
	**Mean PNC Quality Outcomes**						**Difference-in-Differences Estimates**		
	**2010 [Percentage (SD)]**			**2012 Post-Rollout [Percentage (SD)]**			**Arm II** * **Post (SE)**		
**Percent (SD)**	Phase II	Comparison	Diff.	Phase II	Comp-arison	Diff.	**(1)**	**(2)**	**(3)**
**Maternal outcomes**									
Mother received any postnatal checkup	50.4% (2.8%)	58.7% (3.3%)	-8.2%* (4.3%)	53.8% (3.8%)	71.4% (3.8%)	-17.6*** (5.4%)	0.65 (0.3)	0.69 (0.4)	0.74 (0.5)
Mothers who received checkup were seen within 48 hours	75.0% (3.4%)	80.7% (3.4%)	-5.7% (4.9%)	83.5% (3.9%)	77.0% (4.2%)	6.5% (5.8%)	2.12 (1.4)	2.06 (1.3)	2.3 (1.6)
**Newborn outcomes**									
Newborn received any postnatal checkup	97.7% (0.8%)	96.9% (1.2%)	0.82% (1.5%)	96.4% (1.4%)	99.3% (0.7%)	-2.87%* (1.6%)	0.14 (0.2)	0.15 (0.2)	0.14 (0.2)
Newborns who received checkup were seen within 48 hours	59.9% (2.9%)	70.8% (3.1%)	-11.0%*** (4.2%)	73.3% (3.5%)	67.2% (4.0%)	6.14% (5.3%)	2.19* (1.0)	2.25* (1.0)	2.6* (1.5)
**Satisfaction Outcomes**									
Satisfied with services	81.6% (2.2%)	85.2% (2.4%	-3.60% (3.2%)	94.1% (1.8%)	87.1% (2.8%)	6.94%** (3.4%)	3.04* (1.8)	2.85* (1.6)	3.41** (2.0)

*** p<0.01,

** p<0.05,

* p<0.1

Notes: p-values generated using unpaired t-tests assuming unequal variance. “2010” p-values indicate probability of differences between the intervention vs. comparison mean scores at the 2010 data collection time point; “post-rollout” p-values compare the intervention vs. comparison mean sores at the 2012 data collection time point. Difference-in-differences estimates are reported as incidence rate ratios. Robust standard errors are clustered at the health facility level. Columns (1)–(3) report difference-in-difference estimates of program effect under 3 model specifications. Covariates in models (2) and (3) include categorical variables for facility type, facility sector, and client socioeconomic status quintile.

### Subgroup and restricted sample analyses

While equivalence of the comparison and treatment groups was addressed in the sampling phase through the pair-wise matching technique, the lack of randomization in the treatment leads to concerns that the comparison group may not fulfill the parallel trends assumption of the difference-in-differences approach, which says that the estimated causal effect is valid only if the comparison group reflects the PNC quality outcomes that would have been observed in the voucher facilities if the voucher program had not been implemented. We conducted a variety of analyses to check robustness of the sample (S4–S6 Tables). We find that results remain relatively robust across varying levels of facility size and ownership. Greater positive program effects found among higher-level facilities suggests that any bias introduced by differences in facility level across the groups would be expected to result in an underestimate of phase II program effect, as fewer higher-level facilities were represented in the phase II arm. We also present results for phase I program effect excluding Nairobi observations, as no pair-wise county match was selected for Nairobi. Exclusion of the Nairobi sample results program effect estimates that are larger in magnitude and statistical significance than the full sample estimates, again indicating that bias introduced by the Nairobi sample is likely to result in underestimation of program effect on quality of care.

## Discussion

Our findings suggest that participation in Kenya’s reproductive health voucher program since its first phase in 2006 has resulted in significant improvements in quality of postnatal care. We estimate program participation since 2006 has resulted in a 39% overall quality improvement in PNC processes (p = 0.03). Overall quality improvement attributable to the voucher participation appears to be concentrated in care for the mother, with an estimated 86% (FDR q = 0.06) improvement attributed to program participation. We find 5- and 2.5-fold improvements in the counseling on contraceptive methods and postpartum return to fertility attributable to voucher program participation since phase I. These findings indicate that PNC clients at voucher facilities are receiving more comprehensive counseling on postnatal return to fertility, healthy birth spacing, and available contraceptive methods than postnatal clients at comparable non-voucher facilities. Despite improvement in maternal-focused care, we find no significant program effect on the quality of newborn-related components of the postnatal consultation.

These findings are consistent with the existing literature, with Nicaragua’s RH voucher program found to significantly improve aspects of FP counseling and uptake [22]. Program effect on client-reported satisfaction are also consistent with the existing literature. We find two- and three-fold higher odds of clients reporting complete satisfaction with PNC services in phase I and phase II voucher facilities relative to the comparison group, similar to the Nicaraguan voucher program’s finding that voucher users had 2.5 times the odds of reporting satisfaction with RH services as compared to non-voucher users [24].

It is plausible that the voucher program led to significant gains in quality of fertility and FP advice due to increased availability and variety of contraceptives at voucher facilities. As the voucher scheme includes a separate FP voucher that subsidizes long-acting and reversible contraceptives and permanent methods, it might be expected that voucher facilities would be more likely to stock contraceptives. Increased supply of contraceptives could lead to positive spillovers, such as providers more readily discussing and offering these methods to PNC clients. Quality improvements in FP and fertility advice may also be the result of increased knowledge or experience on the part of health care providers in discussing FP and fertility in the postnatal period, if the improved availability of FP methods increases client demand for these services at voucher-contracted facilities. Further research, including longitudinal analysis of provider knowledge and attitudes as well as voucher programs that only accept vouchers for a single service (such as Safe Motherhood but not family planning, or vice versa), is necessary to substantiate these hypotheses and to build empirical evidence regarding the mechanisms through which the voucher program influences quality within each postnatal care domain.

Despite overall improvements in maternal-focused PNC processes, our findings imply that the voucher program may not adequately address key maternal health interventions. We find that a high percentage of women at PNC consultations reported not having received any checkup in the postnatal period. While the mean percentages of women receiving postnatal checkups increased over the study period across all intervention arms, facility participation in the voucher program was not found to have any significant effect on the probability of a woman reporting having received a physical examination in the postnatal period. At 2012 data collection, women in Phase II facilities were in fact significantly less likely to receive a postnatal checkup relative to women at comparison facilities. These findings indicate that the maternal physical examination—a critical component of PNC for identifying complications and preventing maternal morbidity and mortality—is not being adequately addressed through the voucher program as currently implemented.

The lack of significant quality improvements due to voucher program participation in the newborn care processes of PNC is notable. We postulate that the voucher program’s lack of effect on quality of newborn-related PNC may be due to the commonly held opinion in Kenya that PNC is solely focused on the newborn, resulting in higher quality standards for newborn PNC across all facilities [35]. It has been reported that over-worked health providers will often omit the maternal care component of PNC visits entirely [35].

Our findings imply high variability in quality changes in the Phase II facilities, with phase II estimates of voucher program impact lacking statistical significance due to large standard errors. Attenuated program effects are not surprising, given that phase II facilities had been participating in the voucher program for approximately 6 fewer years at post-rollout data collection compared to phase I facilities. High variability in the estimates can be explained in part by the variable implementation of the program across phase II facilities: While the voucher program was rolled out in some phase II facilities immediately after data collection in 2010, others did not begin until mid-2011. Phase II program impact estimates are largest in magnitude for the same components of maternal care with the largest estimated phase I program effects, a consistency that supports the internal and external validity of the estimated phase I program effects.

This paper aims to strengthen the existing evidence base on voucher program’s effectiveness at improving quality of PNC service provision through the use of observed in addition to self-reported quality measures. Program effect estimates on self-reported measures are plausibly biased by differential reporting if voucher users are distinct across a number of unobservable factors as compared to non-voucher users. Voucher users may be expected to be more discerning of quality of care, given their choice to buy a voucher. This may bias estimates of program effect on self-reported satisfaction in the negative direction, if voucher users are more critical of quality of care and thus less likely to report complete satisfaction. As the percentage of voucher users in the 2010 and 2012 samples increases only slightly (from 60.4% in 2010 to 63.7% in 2012), differential reporting would not be expected to introduce large bias into the program effect estimates for self-reported measures in this group. Differential reporting bias is more concerning in the phase II sample, in which the percentage of voucher users increases from 4.5% to 77% from 2010 to 2012. Self-reported outcome measures such as self-reported satisfaction may thus be of limited value in evaluating changes in quality of clinical care. Data on observed clinical process indicators, however, are not subject to such bias.

The analysis is further strengthened by its quasi-experimental design. All identified evaluations of reproductive health voucher program impact on quality have either before-and-after designs without comparison groups or cross-sectional study designs, both of which are subjectively weak designs for causal inference [13–14, 17]. By leveraging two data collection periods and a comparison group, we are able to adjust for time-invariant differences between the intervention and comparison arms, as well as any factors that may change over time but that would be expected to affect all facilities equally (such as general time trends). We are therefore able to avoid a number of possible endogeneity issues, such as temporal trends in quality of PNC across groups,. The presence of multiple rollout years further limits concern that estimated treatment effects are the cause of differential geographic shocks between voucher and comparison counties.

These results are subject to a number of limitations. While the sub-group and restricted sample analyses provide evidence that results are robust across levels of facility ownership and type, there remains the possibility that the trends in quality observed in the comparison group do not accurately represent quality trends that would have been observed in the intervention group had the program not been implemented. In the absence of randomization, it is possible that intervention and comparison facilities experienced differential trends in their quality of care, which violates the difference-in-differences identification assumption. An additional limitation is the lack of a true baseline for the phase I sample, which had been participating in the voucher program for approximately 4 years at the time of the 2010 data collection. We do, however, collect a true baseline for the phase II facilities, and the attenuated results observed in phase II relative to phase I point towards a potential “dose-response” effect of progressive quality improvement with longer program participation. The short period between program implementation in phase II and post-rollout collection presents additional challenges to observing program impact on quality in phase II, particularly if quality improvement mechanisms are ongoing and progressive as has been hypothesized.

Furthermore, although data collected via clinical observation avoids self-report bias and allows collection of more complex clinical information, there is also the possibility of a Hawthorne effect [32, 36]. Hawthorne effects, defined as changes in behavior caused by the act of being observed, would not be expected to bias the difference-in-differences estimates of program effect on PNC processes, as voucher and non-voucher facility consultations were observed under the same protocol. If Hawthorne effects are expected to bias the parameter estimates in voucher and non-voucher groups equally, then this bias should not threaten internal validity of comparisons across groups. However, Hawthorne effects may introduce positive bias into the estimated mean PNC process scores reported in Table 4. The act of being observed may also have influenced quality of care in a number of other ways. For example, permission to proceed with data collection and observation was typically requested through a visit by the study team in advance of the start of data collection. If facilities learned of the date of data collection in advance and shared this information with providers, this could plausibly lead to changes in provider behavior due to the advance warning. Any bias introduced through such changes in provider behavior would be expected to bias the absolute but not the relative magnitude of estimated parameters in the observed PNC process measures.

In spite of these limitations, this study is, to the best of our knowledge, the first to use a strong evaluation design to estimate the causal effects of a RH voucher program on quality of PNC service provision. Its focus on postnatal care strengthens a sparse evidence base on an important but often-neglected intervention point for mothers and newborns.

## Conclusion

Kenya’s RH voucher program has previously been shown to increase access to critical maternal and newborn health services for vulnerable communities. This study strengthens the evidence base for competitive health vouchers as effective mechanisms for quality improvement, and has important policy implications for future scale-up of Kenya’s program and for the design of RH voucher programs more broadly. Despite quality improvement in maternal-focused components of PNC, we find a concerning proportion of mothers seeking PNC are not being checked by any provider after delivery. Maternal care-seeking behavior in the postnatal period appears to be primarily driven by the newborn vaccination schedule or neonatal complications, rather than mothers returning to seek care for themselves [35]. One strategy to reduce current gaps in maternal care is to include requirements for protocols and processes within the quality accreditation process that ensure all mothers delivering in the facility are checked by a skilled provider before discharge, or that otherwise standardize provision of packaged postnatal interventions to both mother and newborn simultaneously [35].

This study addresses an important gap in the existing reproductive health literature by using a strong evaluation design to assess the effectiveness of voucher programs on quality improvement. We find that Kenya’s Safe Motherhood voucher can significantly improve the quality of several key aspects of PNC provision and client-reported outcomes. Out findings further suggest a potential “dose-response” effect of greater quality improvement with longer duration of program participation, which provides support for ongoing voucher schemes. Additional research is needed to quantify the impact of these voucher-driven quality improvements in PNC on maternal and newborn morbidity and mortality as well as population health outcomes.

## Supporting Information

S1 Table Difference-in-differences estimates of program effect on PNC processes—Goodness of fit. (DOCX)

S2 Table Difference-in-differences estimates of program effect on PNC outcomes—Goodness of fit. (DOCX)

S3 Table Ordinary Least Squares difference-in-differences estimates of program effect on PNC processes—Specification check. (DOCX)

S4 Table Sub-group analyses of voucher program impact on PNC process domains. (DOCX)

S5 Table Difference-in-differences estimates adjusted for Integra Initiative participation—Robustness check. (DOCX)

S6 Table Impact of voucher program on PNC processes and outcomes (including and excluding Nairobi)—Robustness check. (DOCX)
