# Supplementary material for: Can Reproductive Health Voucher Programs Improve Quality of Postnatal Care? A Quasi-Experimental Evaluation of Kenya’s Safe Motherhood Voucher Scheme
Source: PLoS One. 2015 Apr 2;10(4):e0122828. doi: 10.1371/journal.pone.0122828 (PMC4383624; doi:10.1371/journal.pone.0122828)
Supplement: S1 Table — (DOCX) [file pone.0122828.s001.docx]

**S1 Table. Difference-in-Differences Estimates of Program effect on PNC Processes – Goodness of Fit**

|  | **Difference-in-Differences Incidence Rate Ratios** | | | |
| --- | --- | --- | --- | --- |
|  | **Arm I * Post (SE)**  **(2)** | **Prob > *X*^2^** | **McFadden’s R^2^** | **Observations** |
| **Maternal care (0-41)** | 1.86** (0.51) | p<0.01 | 0.02 | 1024 |
| **Newborn care (0-15)** | 1.24 (0.19) | p=0.28 | 0.01 | 1024 |
| **Interpersonal skills (0-8)** | 1.20** (0.11) | p<0.01 | 0.02 | 1024 |
| **Overall quality of PNC processes (0-64)** | 1.39** (0.217) | p=0.08 | 0.01 | 1024 |
|  |  |  |  |  |
|  | **Phase II * Post (SE)**  **(2)** | **Prob > *X*^2^** | **McFadden’s R^2^** | **Observations** |
| **Maternal care (0-41)** | 1.49 (0.44) | p<0.01 | 0.01 | 850 |
| **Newborn care (0-15)** | 0.92 (0.15) | p=0.31 | 0.01 | 850 |
| **Interpersonal skills (0-8)** | 0.89 (0.15) | p<0.01 | 0.04 | 850 |
| **Overall quality of PNC processes (0-64)** | 1.09 (0.21) | p<0.01 | 0.01 | 850 |

*** p<0.01, ** p<0.05, * p<0.1

Notes: Negative binomial difference-in-differences estimates are reported as incidence rate ratios. Robust standard errors are clustered at the health facility level. Covariates in model include categorical variables for facility type, facility sector, and client socioeconomic status quintile. The “phase I” and “phase II” covariates are dummies for facility inclusion in phase I and phase II of the voucher program, respectively. “Post” is a time dummy for 2012, with the referent group observations from 2010. The DD estimator is the interaction between the phase (I or II) and post dummies.
