# Supplementary material for: Can Reproductive Health Voucher Programs Improve Quality of Postnatal Care? A Quasi-Experimental Evaluation of Kenya’s Safe Motherhood Voucher Scheme
Source: PLoS One. 2015 Apr 2;10(4):e0122828. doi: 10.1371/journal.pone.0122828 (PMC4383624; doi:10.1371/journal.pone.0122828)
Supplement: S2 Table — (DOCX) [file pone.0122828.s002.docx]

**S2 Table. Difference-in-Differences Estimates of Program Effect on Self-Reported PNC Outcomes – Goodness of Fit**

|  | **Difference-in-Differences Odds Ratios** | | | |
| --- | --- | --- | --- | --- |
|  | **Arm I * Post (SE)** | **Prob > *X*^2^** | **McFadden’s R^2^** | **Observations** |
|  | **(2)** |  |  |  |
| **Maternal outcomes** |  |  |  |  |
| Mother received any postnatal checkup | 1.22 (0.64) | p=0.01 | 0.03 | 1021 |
| Mothers who received checkup seen within 48 hours | 1.70 (1.11) | p<0.01 | 0.06 | 601 |
| **Newborn outcomes** |  |  |  |  |
| Newborn received any postnatal checkup | 0.80 (1.08) | p=0.29 | 0.06 | 930 |
| Newborns who received checkup seen within 48 hours | 1.40 (0.69) | p<0.01 | 0.11 | 905 |
| **Satisfaction Outcomes** |  |  |  |  |
| Satisfied with services | 2.08 (1.24) | p=0.02 | 0.03 | 1019 |
|  | **Arm II * Post (SE)** | **Prob > *X*^2^** | **McFadden’s R^2^** | **Observations** |
| **Percent (SD)** | **(2)** |  |  |  |
| **Maternal outcomes** |  |  |  |  |
| Mother received any postnatal checkup | 0.69 (0.35) | p<0.01 | 0.05 | 848 |
| Mothers who received checkup were seen within 48 hours | 2.08 (1.34) | P=0.22 | 0.02 | 482 |
| **Newborn outcomes** |  |  |  |  |
| Newborn received any postnatal checkup | 0.15 (0.18) | p=0.29 | 0.03 | 830 |
| Newborns who received checkup were seen within 48 hours | 2.29* (1.04) | p<0.01 | 0.07 | 808 |
| **Satisfaction Outcomes** |  |  |  |  |
| Satisfied with services | 2.87* (1.61) | p<0.01 | 0.06 | 847 |

*** p<0.01, ** p<0.05, * p<0.1

Notes: Logistic regression results presented as odds ratios. Covariates in model include categorical variables for facility type, facility sector, and client socioeconomic status quintile. The “phase I” and “phase II” covariates are dummies for facility inclusion in phase I and phase II of the voucher program, respectively. “Post” is a time dummy for 2012, with the referent group observations from 2010. The DD estimator is the interaction between the phase (I or II) and post dummies.
