# Supplementary material for: Can Reproductive Health Voucher Programs Improve Quality of Postnatal Care? A Quasi-Experimental Evaluation of Kenya’s Safe Motherhood Voucher Scheme
Source: PLoS One. 2015 Apr 2;10(4):e0122828. doi: 10.1371/journal.pone.0122828 (PMC4383624; doi:10.1371/journal.pone.0122828)
Supplement: S3 Table — (DOCX) [file pone.0122828.s003.docx]

**S3 Table. Ordinary Least Squares Difference-in-Differences Estimates of Program Effect on PNC Processes –**

**Specification Check**

|  | **OLS Difference-in-Differences Estimator** | | | |
| --- | --- | --- | --- | --- |
|  | **Arm I * Post (SE)**  **(2)** | **R^2^** | **Prob > F** | **Observations** |
| **Maternal care** |  |  |  |  |
| History taking practices (0-7) | 0.84* (0.44) | 0.09 | p<0.01 | 1024 |
| Physical examination of the mother (0-7) | 0.44 (0.43) | 0.03 | p=0.03 | 1024 |
| Advice on danger signs for the mother (0-3) | 0.07 (0.14) | 0.02 | p=0.03 | 1024 |
| Fertility advice (0-4) | 0.77** (0.31) | 0.08 | p<0.01 | 1024 |
| Family planning methods discussed (0-10) | 0.84** (0.40) | 0.06 | p<0.01 | 1024 |
| STI/HIV risk assessment (0-3) | 0.10 (0.11) | 0.03 | p=0.41 | 1024 |
| STI/HIV risk factors (0-4) | 0.16 (0.13) | 0.04 | p=0.15 | 1024 |
| STI management (0-3) | 0.02 (0.03) | <0.01 | . | 1024 |
| **Total for maternal care (0-41)** | 3.24** (1.59) | 0.09 | p<0.01 | 1024 |
| **Newborn care** |  |  |  |  |
| Newborn feeding advice (0-3) | 0.63 (0.42) | 0.07 | p=0.06 | 1024 |
| Newborn examination (0-4) | 0.20 (0.25) | 0.06 | p=0.04 | 1024 |
| Newborn danger signs discussed (0-4) | 0.17 (0.24) | 0.07 | p<0.01 | 1024 |
| Documentation (0-4) | 0.39 (0.41) | 0.06 | p=0.07 | 1024 |
| **Total for newborn care (0-15)** | 1.39 (0.94) | 0.05 | p=0.35 | 1024 |
| **Interpersonal skills** |  |  |  |  |
| Creation of rapport (0-8) | 0.77* (0.39) | 0.13 | p<0.01 | 1024 |
|  |  |  |  |  |
| **Overall (0-64)** | 5.40** (2.46) | 0.07 | p=0.08 | 1024 |
|  |  |  |  |  |
|  | **Phase II * Post (SE)**  **(2)** | **R^2^** | **F-Test** | **Observations** |
| **Maternal care** |  |  |  |  |
| History taking practices (0-7) | 1.03** (0.42) | 0.11 | p<0.01 | 850 |
| Physical examination of the mother (0-7) | -0.03 (0.40) | 0.04 | p=0.14 | 850 |
| Advice on danger signs for the mother (0-3) | -0.13 (0.12) | 0.04 | p=0.18 | 850 |
| Fertility advice (0-4) | 0.39 (0.41) | 0.04 | p=0.25 | 850 |
| Family planning methods discussed (0-10) | 0.85 (0.65) | 0.06 | p<0.1 | 850 |
| STI/HIV risk assessment (0-3) | 0.17 (0.13) | 0.03 | p=0.33 | 850 |
| STI/HIV risk factors (0-4) | 0.25 (0.17) | 0.04 | p=0.54 | 850 |
| STI management (0-3) | -0.10 (0.07) | 0.04 | p=0.69 | 850 |
| **Total for maternal care (0-41)** | 2.43 (1.67) | 0.05 | p<0.01 | 850 |
| **Newborn care** |  |  |  |  |
| Newborn feeding advice (0-3) | 0.31 (0.50) | 0.09 | p<0.1 | 850 |
| Newborn examination (0-4) | -0.21 (0.25) | 0.05 | p=0.10 | 850 |
| Newborn danger signs discussed (0-4) | -0.43* (0.25) | 0.04 | p<0.01 | 850 |
| Documentation (0-4) | -0.22 (0.44) | 0.05 | p=0.72 | 850 |
| **Total for newborn care**  **(0-15)** | -0.55 (0.98) | 0.03 | p=0.25 | 850 |
| **Interpersonal skills** |  |  |  |  |
| Creation of rapport (0-8) | -0.41 (0.70) | 0.18 | p<0.1 | 850 |
|  |  |  |  |  |
| **Overall (0-64)** | 1.46 (3.01) | 0.05 | p<0.1 | 850 |

*** p<0.01, ** p<0.05, * p<0.1

Notes: Difference-in-differences estimates reported use the Ordinary Least Squares (OLS) estimator. Robust standard errors are clustered at the health facility level. Covariates in model include categorical variables for facility type, facility sector, and client socioeconomic status quintile. The “phase I” and “phase II” covariates are dummies for facility inclusion in phase I and phase II of the voucher program, respectively. “Post” is a time dummy for 2012, with the referent group observations from 2010. The DD estimator is the interaction between the phase (I or II) and post dummies.
