# Supplementary material for: Can Reproductive Health Voucher Programs Improve Quality of Postnatal Care? A Quasi-Experimental Evaluation of Kenya’s Safe Motherhood Voucher Scheme
Source: PLoS One. 2015 Apr 2;10(4):e0122828. doi: 10.1371/journal.pone.0122828 (PMC4383624; doi:10.1371/journal.pone.0122828)
Supplement: S4 Table — (DOCX) [file pone.0122828.s004.docx]

**S4 Table. Sub-Group Analyses of Voucher Program Impact on PNC Process Domains**

|  | **Phase I vs. Comparison Group** | | | | | | |
| --- | --- | --- | --- | --- | --- | --- | --- |
|  |  |  |  |  |  |  |  |
|  | **Phase I * Post** | | | | | | |
|  | **All facilities** | | Only higher-level facilities (hospital, sub-district hospitals) | | Only lower-level facilities (health centers, dispensaries, clinics and nursing homes) | Only public facilities | Only private facilities |
| Overall maternal care score | **1.86** (0.5)** | | 1.82 (0.7) | | 2.01** (0.7) | 1.68 (0.6) | 2.20* (0.9) |
| Overall newborn care score | **1.24 (0.2)** | | 1.25 (0.2) | | 1.29 (0.3) | 1.32* (0.2) | 1.06 (0.3) |
| Interpersonal care score | **1.20** (0.1)** | | 1.24* (0.1) | | 1.16 (0.2) | 1.14 (0.1) | 1.29** (0.1) |
| Overall process score | **1.39** (0.2)** | | 1.41 (0.3) | | 1.46 (0.3) | 1.38* (0.3) | 1.38 (0.3) |
|  |  | |  | |  |  |  |
|  | | **Phase II vs. Comparison Group** | | | | | |
|  | |  |  |  |  |  |  |
|  | | **Phase II * Post** | | | | | |
|  | | **All facilities** | | Only higher-level facilities (hospital, sub-district hospitals) | Only lower-level facilities (health centers, dispensaries, clinics and nursing homes) | Only public facilities | Only private facilities |
| Overall maternal care score | | **1.49 (0.4)** | | 2.62*** (0.8) | 0.88 (0.3) | 1.78 (0.6) | 1.09 (0.3) |
| Overall newborn care score | | **0.92 (0.1)** | | 1.00 (0.3) | 0.87 (0.1) | 0.98 (0.2) | 0.72** (0.1) |
| Interpersonal care score | | **0.89 (0.2)** | | 1.07 (0.3) | 0.80 (0.1) | 0.92 (0.2) | 0.84** (0.1) |
| Overall process score | | **1.09 (0.2)** | | 1.43 (0.4) | 0.86 (0.2) | 1.20 (0.3) | 0.84 (0.1) |

*** p<0.01, ** p<0.05, * p<0.1.

Notes: Results reported as odds ratios; robust standard errors clustered at the health facility level. Models include categorical variables for facility type and sector and client socioeconomic status quintile. DD estimator is the interaction between phase and post dummies.
