# Supplementary material for: Can Reproductive Health Voucher Programs Improve Quality of Postnatal Care? A Quasi-Experimental Evaluation of Kenya’s Safe Motherhood Voucher Scheme
Source: PLoS One. 2015 Apr 2;10(4):e0122828. doi: 10.1371/journal.pone.0122828 (PMC4383624; doi:10.1371/journal.pone.0122828)
Supplement: S5 Table — (DOCX) [file pone.0122828.s005.docx]

**S5 Table. Difference-in-Differences Estimates Adjusted for Integra Initiative Participation – Robustness Check**

|  | **Phase I** | |  | **Phase II** | |
| --- | --- | --- | --- | --- | --- |
|  |  |  |  |  |  |
|  | **Phase I*Post + covariates** | **Phase I*Post + covariates + Integra dummy** |  | **Phase II*Post + covariates** | **Phase II*Post + covariates + Integra dummy** |
| **Process** |  |  |  |  |  |
| Maternal care | 1.86** (0.5) | 1.90** (0.5) |  | 1.49 (0.4) | 1.45 (0.5) |
| Newborn care | 1.24 (0.2) | 1.25 (0.2) |  | 0.92 (0.2) | 0.90 (0.1) |
| Interpersonal skills | 1.20** (0.1) | 1.21** (0.1) |  | 0.89 (0.2) | 0.87 (0.2) |
| Overall process | 1.39** (0.2) | 1.41** (0.2) |  | 1.09 (0.2) | 1.07 (0.2) |
|  |  |  |  |  |  |
| **Outcome** |  |  |  |  |  |
| Mother received postnatal checkup | 1.22 (0.6) | 1.21 (0.6) |  | 0.69 (0.4) | 0.67 (0.3) |
| Mothers who received checkup seen within 48 hours | 1.70 (1.1) | 1.70 (1.1) |  | 2.06 (1.3) | 2.07 (1.3) |
| Newborn received postnatal checkup | 0.80 (1.1) | 0.78 (1.1) |  | 0.15 (0.2) | 0.14 (0.2) |
| Newborns who received checkup seen within 48 hours | 1.40 (0.7) | 1.39 (0.7) |  | 2.25* (1.0) | 2.27* (1.0) |
| Satisfied with services | 2.08 (1.2) | 2.09 (1.2) |  | 2.85* (1.6) | 2.84* (1.6) |

*** p<0.01, ** p<0.05, * p<0.1.

Notes: Results are reported as incidence rate ratios. Robust standard errors are clustered at the health facility level. The baseline model includes categorical variables for intervention time (pre-post), intervention arm (phase I), facility type, facility sector, and client socioeconomic status quintile. The difference in difference estimator is the interaction between the phase (I or II) and post dummies. A possible threat to the difference-in-differences identification strategy would be the presence of other programs or policies that may differentially impact intervention and comparison facilities over the course of the study period, such as the Integra Initiative [35]. A research effort that began in 2010 to strengthen empirical evidence for integration of RH and HIV services, Integra was implemented in select public facilities across phase I (1 facility) and phase II (2 facilities). While it remains possible that differential inputs due to Integra over the study period bias estimates, this probability is reduced by the high consistency of the results before and after adjustment for Integra participation (Table 6).
