# Supplementary material for: Can Reproductive Health Voucher Programs Improve Quality of Postnatal Care? A Quasi-Experimental Evaluation of Kenya’s Safe Motherhood Voucher Scheme
Source: PLoS One. 2015 Apr 2;10(4):e0122828. doi: 10.1371/journal.pone.0122828 (PMC4383624; doi:10.1371/journal.pone.0122828)
Supplement: S6 Table — (DOCX) [file pone.0122828.s006.docx]

**S6 Table. Impact of Voucher Program on PNC Processes and Outcomes (Including and Excluding Nairobi) –**

**Robustness Check**

|  | **Phase I vs. Comparison Facilities** | | | | | | | |
| --- | --- | --- | --- | --- | --- | --- | --- | --- |
|  | **Phase I*Post** | | |  | | **Phase I*Post + covariates** | | |
| **Maternal Care Quality Scores** | **Phase I Full Sample** | **Excl. Nairobi** | |  | | **Phase I Full Sample** | | **Excl. Nairobi** |
| History taking practices (0-7) | 1.48* (0.3) | 1.74** (0.4) | |  | | 1.47* (0.3) | | 1.75** (0.4) |
| Maternal physical exam (0-7) | 1.59 (0.6) | 1.94 (0.8) | |  | | 1.51 (0.6) | | 1.88 (0.7) |
| Maternal danger signs advice (0-3) | 1.60 (1.3) | 1.85 (1.6) | |  | | 1.53 (1.2) | | 1.80 (1.5) |
| Fertility advice (0-3) | 2.64** (1.0) | 2.92** (1.3) | |  | | 2.58** (1,0) | | 2.96*** (1.2) |
| Family planning methods discussion (0-10) | 3.40** (1.7) | 3.70** (2.0) | |  | | 5.01*** (2.9) | | 4.81** (3.0) |
| STI/HIV risk assessment (0-3) | 3.45 (3.2) | 5.22* (5.2) | |  | | 3.81 (3.4) | | 8.29** (7.4) |
| STI/HIV risk factors (0-3) | 2.52 (1.8) | 2.99 (2.2) | |  | | 2.53 (1.6) | | 3.46* (2.4) |
| STI management (0-3) | 3.36 (4.4) | 2.96 (4.0) | |  | | 3.44 (4.3) | | 2.64 (3.3) |
| **Total maternal care score (0-41)** | **1.92** (0.5)** | **2.25*** (0.7)** | |  | | **1.86** (0.5)** | | **2.31*** (0.7)** |
|  |  |  | |  | |  | |  |
| **Newborn Care Quality Scores** |  |  | |  | |  | |  |
| Newborn feeding advice (0-3) | 1.52 (0.4) | 1.79* (0.5) | |  | | 1.46 (0.4) | | 1.74* (0.5) |
| Newborn physical exam (0-4) | 1.20 (0.3) | 1.39 (0.3) | |  | | 1.17 (0.3) | | 1.37 (0.3) |
| Newborn danger signs advice (0-4) | 1.80 (1.1) | 1.66 (0.9) | |  | | 1.05 (0.6) | | 1.02 (0.6) |
| Documentation (0-4) | 1.11 (0.2) | 1.16 (0.2) | |  | | 1.13 (0.2) | | 1.18 (0.2) |
| **Total newborn care score (0-15)** | **1.27 (0.2)** | **1.38** (0.2)** | |  | | **1.24 (0.2)** | | **1.37** (0.2)** |
|  |  |  | |  | |  | |  |
| **Interpersonal Skills Quality Score** |  |  | |  | |  | |  |
| Creation of rapport (0-8) | **1.20* (0.1)** | **1.31*** (0.1)** | |  | | **1.20** (0.1)** | | **1.31*** (0.1)** |
|  |  |  | |  | |  | |  |
| **Overall Process Score (0-64)** | **1.43** (0.2)** | **1.60*** (0.3)** | |  | | **1.39** (0.2)** | | **1.58*** (0.3)** |
| Observations | 1024 | 888 | |  | | 1024 | | 888 |
|  |  |  | |  | |  | |  |
|  | **Phase I vs. Comparison Facilities** | | | | | | | |
|  | **Phase I*Post** | |  | | **Phase I*Post + covariates** | | | |
| **Maternal Outcomes** | **Phase I Full Sample** | **Excl. Nairobi** |  | | **Phase I Full Sample** | | **Excl. Nairobi** | |
| Mother received any postnatal checkup | 1.20 (0.6) | 1.29 (0.7) |  | | 1.22 (0.6) | | 1.30 (0.7) | |
| Mothers who received checkup were seen within 48 hours | 1.76 (1.2) | 1.84 (1.3) |  | | 1.70 (1.1) | | 1.78 (1.2) | |
|  |  |  |  | |  | |  | |
| **Newborn Outcomes** |  |  |  | |  | |  | |
| Newborn received any postnatal checkup | 0.72 (1.0) | 1.92 (3.0) |  | | 0.80 (1.1) | | 2.10 (3.2) | |
| Newborns who received checkup were seen within 48 hours | 1.53 (0.7) | 1.77 (0.9) |  | | 1.40 (0.7) | | 1.55 (0.8) | |
|  |  |  |  | |  | |  | |
| **Satisfaction Outcomes** |  |  |  | |  | |  | |
| Satisfied with services | 2.03 (1.2) | 3.43* (2.3) |  | | 2.08 (1.2) | | 3.45* (2.3) | |
|  |  |  |  | |  | |  | |

*** p<0.01, ** p<0.05, * p<0.1.

Notes: 138 observations from Nairobi phase I facilities were dropped to create the Nairobi-excluded sample. Robust standard errors are clustered at the health facility level. DD estimates are presented as incidence rate ratios using the negative binomial estimator.
